# Identification of biomarkers, immune infiltration landscape, and treatment targets of ischemia–reperfusion acute kidney injury at an early stage by bioinformatics methods

**DOI:** 10.1186/s41065-022-00236-x

**Published:** 2022-06-04

**Authors:** Ruilian You, Zhige Heyang, Yixin Ma, Peng Xia, Hua Zheng, Jianfeng Lin, Peili Ji, Limeng Chen

**Affiliations:** 1 Department of Nephrology, State Key Laboratory of Complex Severe and Rare Diseases, Peking Union Medical College Hospital, Chinese Academy of Medical Science and Peking Union Medical College, Beijing, 100730 China; 2 Department of Medical Research Center, Peking Union Medical College Hospital, Chinese Academy of Medical Science and Peking Union Medical College, Beijing, 100730 China

**Keywords:** Ischemia/reperfusion injury, Acute kidney injury, Robust rank aggregation, Immune infiltration, Drug-gene interaction network

## Abstract

**Background:**

Mechanisms underlying ischemia/reperfusion injury-acute kidney injury (IRI-AKI) are not fully elucidated. We conducted an integrative analysis of IRI-AKI by bioinformatics methods.

**Methods:**

We screened gene expression profiles of the IRI-AKI at early phase from the Gene Expression Omnibus (GEO) database. Differentially expressed genes (DEGs) were identified and enrichment pathways were conducted based on gene ontology (GO), Kyoto Encyclopedia of Genes and Genomes (KEGG) database, and Gene set enrichment analysis (GSEA). Immune cell infiltration analysis was performed to reveal the change of the microenvironment cell types. We constructed protein–protein interaction (PPI), and Cytoscape with plug-ins to find hub genes and modules. We performed robust rank aggregation (RRA) to combine DEGs and analyzed the target genes for miRNA/transcription factor (TF) and drug-gene interaction networks.

**Results:**

A total of 239 and 384 DEGs were identified in GSE87024 and GSE34351 separately, with the 73 common DEGs. Enrichment analysis revealed that the significant pathways involve mitogen-activated protein kinase (MAPK) signaling, interleukin-17, and tumor necrosis factor (TNF) signaling pathway, etc. RRA analysis detected a total of 27 common DEGs. Immune cell infiltration analysis showed the plasma cells reduced and T cells increased in IRI-AKI. We identified JUN, ATF3, FOS, EGR1, HMOX1, DDIT3, JUNB, NFKBIZ, PPP1R15A, CXCL1, ATF4, and HSPA1B as hub genes. The target genes interacted with 23 miRNAs and 116 drugs or molecular compounds such as curcumin, staurosporine, and deferoxamine.

**Conclusion:**

Our study first focused on the early IRI-AKI adopting RRA analysis to combine DEGs in different datasets. We identified significant biomarkers and crucial pathways involved in IRI-AKI and first construct the immune landscape and detected the potential therapeutic targets of the IRI-AKI by drug-gene network.

**Supplementary Information:**

The online version contains supplementary material available at 10.1186/s41065-022-00236-x.

## Background

Acute kidney injury (AKI), characterized by a rapid decrease in glomerular filtration rate, is a universal disease in hospital with high morbidity and mortality. It is reported that the incidence of AKI is 10–15% of all hospitalizations [1] and approximately 50% in the intensive care unit [2]. The ischemia–reperfusion injury (IRI) is the most common cause of AKI [3], which often occurs after surgery and contributes to adverse outcomes in kidney transplantation. The mismatch between supply and demand of oxygen is the central pathophysiology of the IRI/AKI leading to oxidative metabolism reduction and further injury of tubular epithelial cells [4]. Though several biomarkers, such as kidney injury molecule-1 (KIM-1), neutrophil gelatinase-associated lipocalin (NGAL), and interleukin-18 (IL-18), have been studied for a long time, no one can substitute the creatinine in the clinical setting since low specificity to predict and diagnose AKI. Scientists haven't found pharmacological therapy to prevent or reverse the damage once kidney injury is established. Renal replacement therapy is the only alternative treatment available for severe AKI patients currently [5]. An in-depth understanding of the molecular and cellular pathophysiological mechanisms underlying IRI-AKI will contribute to exploring a more precision approach to detect and treat kidney injury.

Microarray, a high-throughput tool for powerfully performing global gene expression profiles. At present, many studies have applied microarray to explore potential biomarkers and pathways in disease development [6, 7], which provides instructions for further experiments. Since seldom IRI-AKI patients receive kidney biopsy, human kidney specimen is hard-acquired in genome-wide transcriptional analysis. We investigated the transcriptional pathogenesis and progressions of IRI-AKI based on the data from experimental animal models which were widely used in this field.

**Fig. 1:**
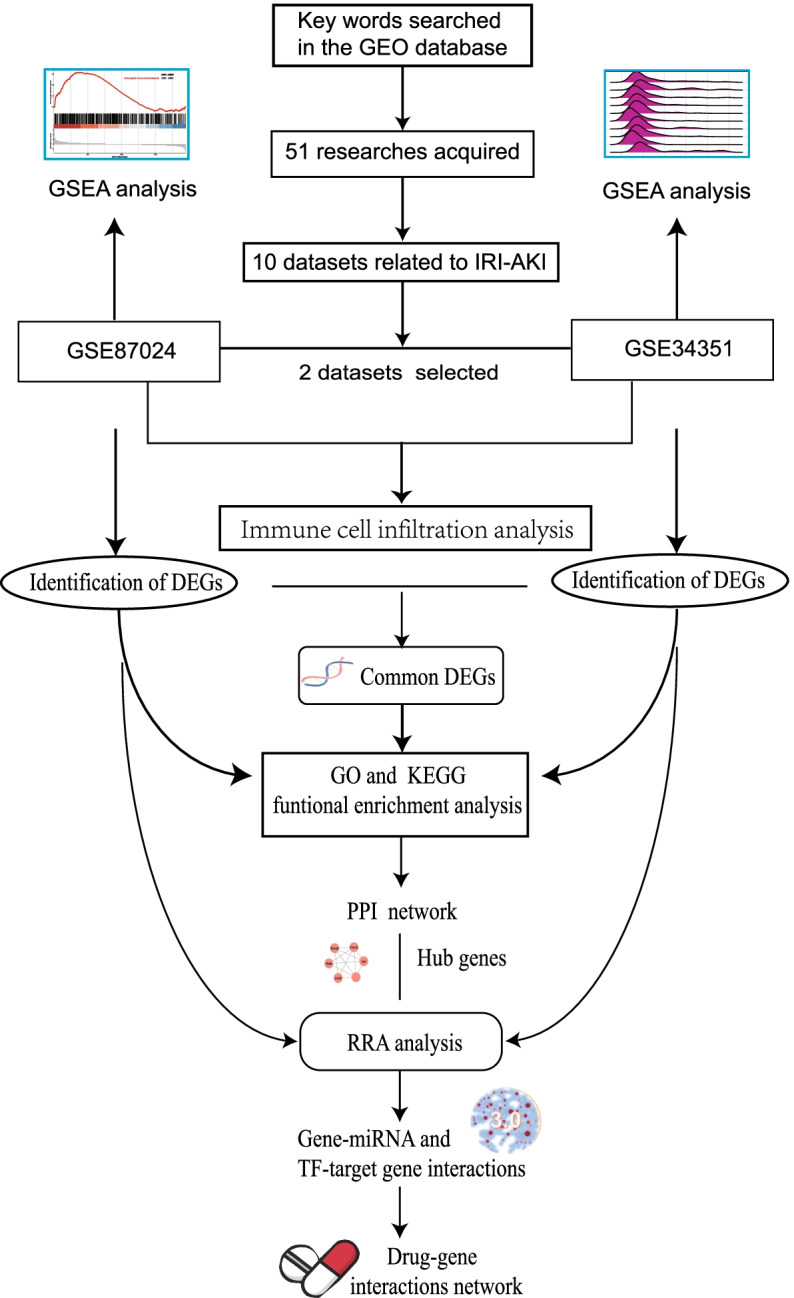
Flow chart of the study design. GEO: Gene Expression Omnibus; GO: gene ontology; KEGG: Kyoto Encyclopedia of Genes and Genomes; GSEA: gene set enrichment analysis; IRI-AKI: Ischemia–reperfusion injury induced acute kidney injury. PPI: Protein–protein interaction; DEGs: differentially expressed genes; WT: wild type

With the development of bioinformatics, several methods have been applied to screen the key biomarkers and pathways involved in the IRI-AKI. However, limited sample sizes of individual studies and the use of different technological platforms lead to substantial inter-study variability. The robust rank aggregation (RRA) is an effective method to integrate differentially expressed genes (DEGs) lists of different platforms, which is both computationally efficient and statistically stable. This method has been used in many disorders, such as gastric cancer [8], papillary thyroid carcinoma [9], and diabetic nephropathy [10], but hasn’t been applied in IRI-AKI thus far. Here, we extracted the samples with the same tissue type and similar genetic background IRI-AKI mice from GSE87024 and GSE34351 datasets in GEO database. We adopted the RRA method to find common DEGs and gene pathways. Further protein–protein interaction (PPI), gene-miRNA/transcription factor (TF) network, and drug-gene interaction network were performed to improve the in-depth understanding of the IRI-AKI (Fig. 1).

## Materials and methods

### Microarray data and normalization

We searched the "acute kidney injury [MeSH Terms] OR acute kidney failure [All fields] OR "renal ischemia–reperfusion injury" OR "ischemic AKI" AND ‘Expression profiling by array’[Filter])" in the Gene Expression Omnibus (GEO) (http://www.ncbi.nlm.nih.gov/geo). The inclusion is: (1) The study focused on the ischemia–reperfusion injury. (2) The sample tissue is the kidney. (3) The organism is wild-type mice. (4) The IRI-AKI time is early within 24 h. Besides, considering detecting the biomarkers of AKI as early as possible and reducing the heterogeneity of different datasets, we chose the two datasets (GSE87024 and GSE34351 published in high-level journals without being analyzed well before (Table 1). For GSE87024, we extracted the IRI-6 h (GSM2319037, GSM2319038, GSM2319039) and sham group (GSM2319034, GSM2319035, GSM2319036) and for GSE34351, we chose the IRI-4 h (n = 3, GSM847661, GSM847662, GSM847663) and control group of wild-type mice (*n* = 3, GSM847664, GSM847665, GSM847666). The method of performing the IRI-AKI model in the dataset GSE34351 was clipping the left renal for either 16 min or 23 min after right nephrectomy, which was like the dataset GSE87024 making the left renal occluded for 17.5 min.

**Table 1:** Characteristics of the individual studies

GEO ID	Platform	Published Time	Organism	Strain	Tissue Type	IRI time	Sample size	Citation (PMID)	Citation (Journal)
GSE87024	GPL6887	2016	Mus musculus	C57BL/6	IRI vs Sham kidney	6 h	3 vs 3	26,823,548	JASN
GSE34351	GPL1261	2012	Mus musculus	C57BL/10	IRI vs Sham kidney	4 h	3 vs 3	22,895,517	KI

*GEO* Gene Expression Omnibus, *IRI* Ischemic renal injury

*JASN* Journal of American Society of Nephrology, *KI* Kidney International

The platform for GSE87024 is GPL6887, Illumina MouseWG-6 v2.0 expression beadchip, while GSE34351 is GPL1261 [Mouse430_2], Affymetrix Mouse Genome 430 2.0 Array. Normalization of these data was carried out with the "limma" R package.

### Identification of differentially expressed genes

We applied the linear model and empirical Bayes model analysis by the "limma" R package to find the DEGs and calculate the differential expression. The |log2fold change (FC)|> 1.5 and *p*-value < 0.05 were used as the significant criteria. Heatmaps and volcano plots of DEGs were conducted using the "Pheatmap" and "ggplot2" packages in R. 4.0.0. An unsupervised principal component analysis (PCA) method was performed to extract two features from each group. The overlapping DEGs were further visualized by the "VennDiagram" R package.

### Functional and pathway enrichment analysis

We conducted the Gene ontology (GO) terms and Kyoto Encyclopedia of Genes and Genomes (KEGG) pathway enrichment analysis of DEGs in different datasets. GO analysis can find the biological characteristics in the biological process (BP) of the genes. KEGG analysis offers a comprehensive knowledge of bio-interpretation of cellular processes and identifies shared pathways of co-expressed genes. We completed and visualized the analysis by the "ClusterProiler" V3.16.1 package [11] (significant criteria is *p* < 0.05 and q-value < 0.05) and "DOSE" v3.16.0 [12].

### Gene set enrichment analysis (GSEA) of the two expression data sets

GSEA is a powerful analytical method to identify whole gene sets, which share common chromosomal location, biological function, or regulation by comparison with knowledge-based databases accumulating large-scale expression data sets [13]. We conducted the GSEA of the two datasets in GO and KEGG respectively. Each analysis performed 1000 times of arrangement of the gene set. The criteria of the significantly enriched gene sets were *P*-value < 0.05. The GSEA analysis was performed by the "ClusterProiler" V3.16.1 package.

### Evaluation of immune cell infiltration

To evaluate the immune cell role and change in the IRI-AKI, we conducted the immune cell infiltration analysis by CIBERSORT method [14]. CIBERSORT can accurately estimate the immune composition of tissue. We conducted this analysis by " CIBERSORT.R" script and visualized the results by "pheatmap" and "ggpubr" R packages.

### Construction and analysis of protein and protein interactions (PPI) network

We searched the common DEGs in the online tool STRING (http://www.string-db.org/) to construct the PPI network showing interactions between genes or proteins. We conducted the confidence score of 0.4 as the cut-off value, visualized the PPI network of DEGs by Cytoscape software [15]. Cytohubba and CytoNCA [16] plug-in were employed to identify the hub genes separately. We adopted 11 methods (MCC displays a satisfying comparative performance) in Cytohubba and 3 evaluation indexes (degree centrality (DC), betweenness centrality (BC), closeness centrality (CC) applied in the CytoNCA. We further extracted hub modules using another plug-in - Molecular Complex Detection (MCODE) with the cut-off score of 2.

### Robust rank aggregation (RRA) method to find the DEGs

We used the de-bach effect, the robust rank aggregation, and probabilistic models to integrate sorted lists of different gene expression profiles from the different protocols or measurement platforms. Based on each gene freely arranged in each data assumption, we scored the rank vector by the order-based statistical analysis and defined the final score of each vector as the minimum *p*-value. The *p*-value is corrected to determine whether the ranking of a specific gene is statistically significant, and multiple checks to assess the stability of the acquired *p* values. We repeated leave-one-out cross-validation 10,000 times and calculated the averaged *p* values from all rounds. If a gene ranks high in the results of all DEGs, the smaller *p*-value was by the RRA method and with the greater probability of the DEGs' robustness. This process was conducted by the “RobustRankAggreg” R package [17].

### Transcription factor (TF)-gene interactions and gene-miRNA network of the combined DEGs and hub genes

We constructed the gene-miRNA network and TF-gene interactions of the combined DEGs from RRA analysis and hub genes in the NetworkAnalyst [18] (https://www.net workanalyst.ca/), which is a comprehensive web platform for gene expression analysis. The gene-miRNA network is based on the miRTarBase (http://mirtarbase.mbc.nctu.edu. tw/php/download.php), while TF-gene interactions based on the ENCODE (http://cistrome.org/ BETA/).

### Construction of Drug Gene Interaction network

The Drug Gene Interaction Database (DGIdb) version 3.0.2 (https://www.dgidb.org) consolidates, synthesis, and normalizes drug-gene interactions and gene druggability information from 30 disparate sources [19]. We searched the DEGs genes from the RRA analysis and hub genes in the DGIdb to explore potential drugs or molecular compounds that interacted with the genes. The drug-gene interaction network was visualized by the Cytoscape software.

## Results

### Identification of differentially expressed genes

**Fig. 2:**
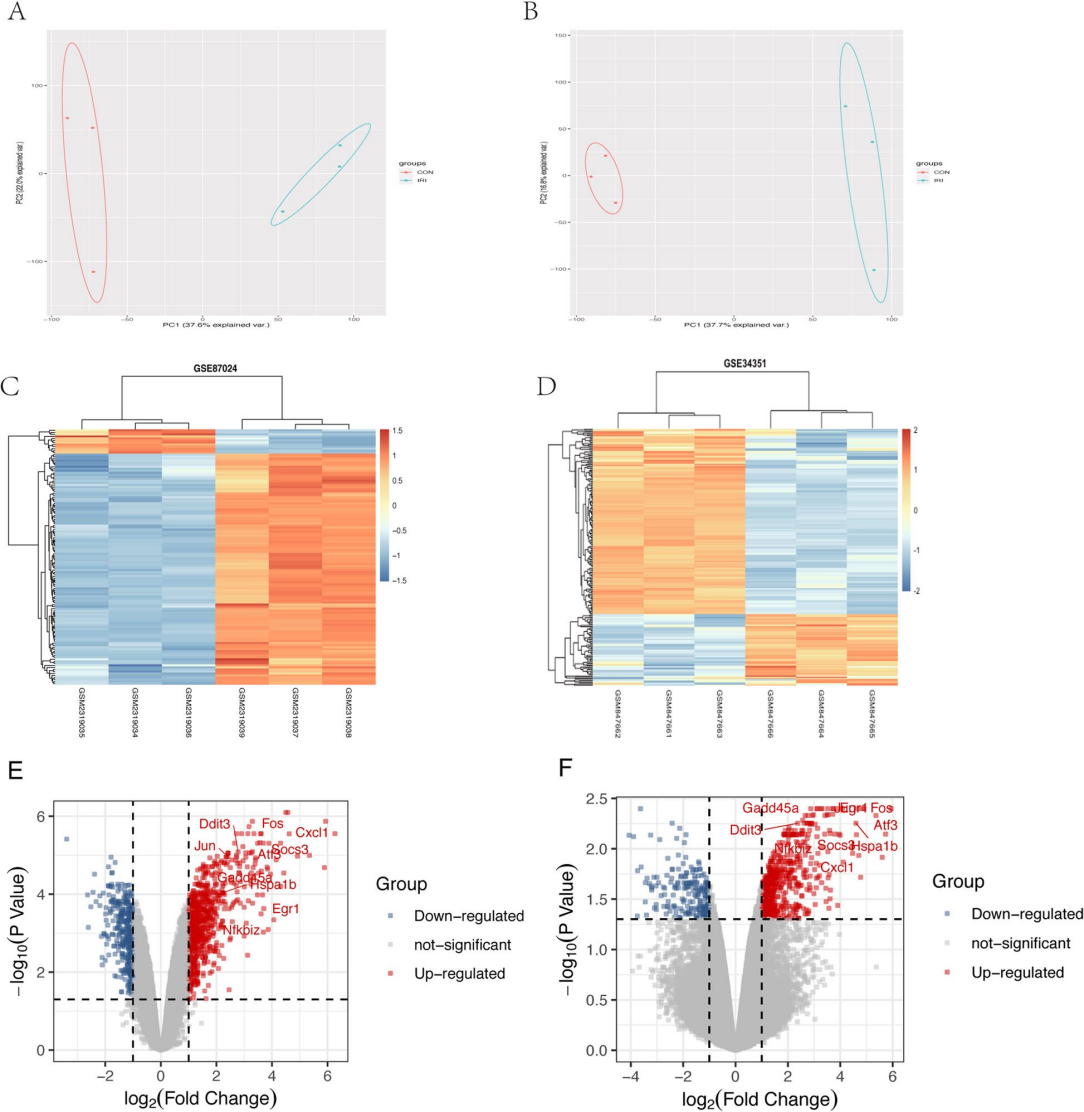
Principal components analysis (PCA) score trajectory plots, heatmaps, and volcano plots of the two datasets. **A**, **B** Principal components analysis (PCA) score trajectory plots (A: GSE87024; B: GSE34351) indicate obvious differences between the ischemic acute kidney injury (IRI) and control (CON) group. **C**, **D** Heatmaps and showing the differentially expressed genes (DEGs) between the IRI and CON group. The red color represents the up-regulated genes, while the blue color represents the down-regulated genes. Samples are sorted by columns, and genes are sorted by rows. **E**, **F** Volcano plots showed the significantly DEGs in two datasets (A: GSE87024; B: GSE34351). Red points represent up-regulated, and green represent down-regulated genes. The differences are set as |log FC|> 1.5 and *P* < 0.05

After standardization of the two datasets (Figure S1), 239 (187 up-regulated and 52 down-regulated genes) and 384 DEGs (259 up-regulated and 125 down-regulated genes) were extracted from the GSE87024 and GSE34351. PCA score trajectory plots indicated that the IRI and CON groups didn't overlap suggesting the apparent differences between the two groups (Fig. 2 A-B). Heatmaps showed the DEGs could discriminate between the IRI and control (CON) groups (Fig. 2 C-D). The volcano plots visualized the distribution of DEGs (Fig. 2E-F).

### Functional and pathway enrichment analysis

**Fig. 3:**
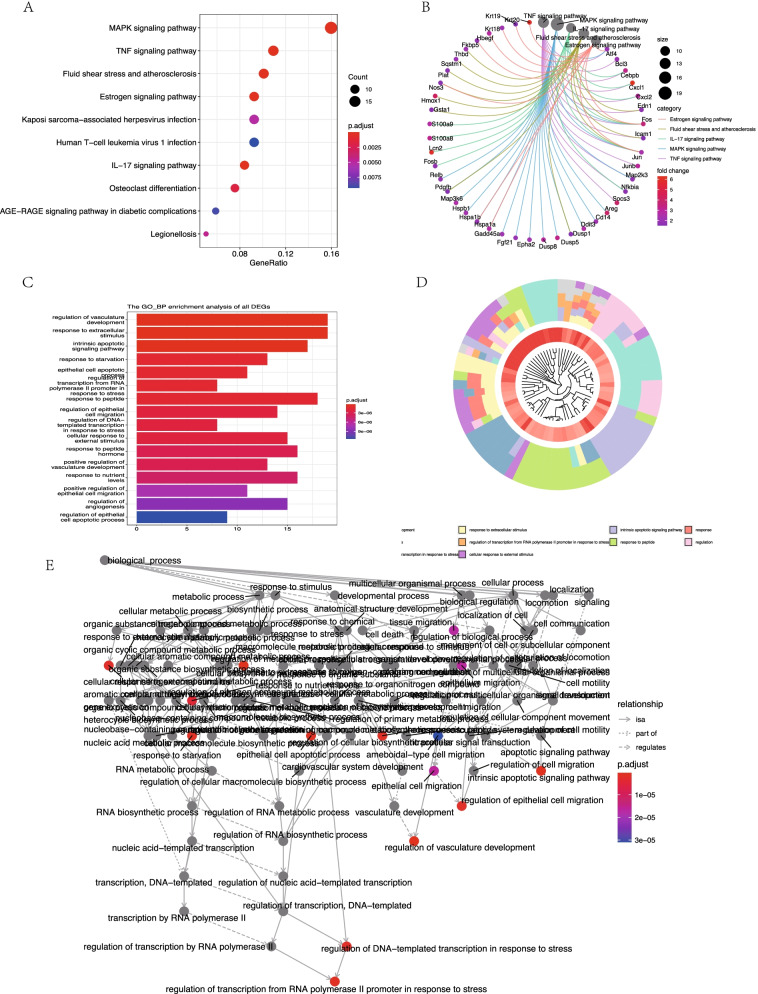
Gene Ontology (GO) and Kyoto Encyclopedia of Genes and Genomes analysis (KEGG) enrichment of up-regulated differentially expressed genes (DEGs) in GSE87024. **A** Advanced bubble chart shows significant KEGG pathways of the DEGs. **B** Cneplot visualized the conjunction between genes and the enrichment pathway. **C** Bar chart visualized the GO enrichment significance items of DEGs. **D** GO Cluster plot showed the interaction between clusters and genes in GO terms. **E** GO plot of the interactions between different GO terms

For up-regulated genes in GSE87024, the KEGG pathway analysis acquired the 23 significant pathways with the top 3 pathways are TNF signaling pathway, MAPK signaling pathway, and IL-17 signaling pathway (Fig. 3A). Cneplot visualized the conjunction between genes and the enrichment pathway(Fig. 3B). GO functional enrichment analysis showed up-regulated genes were mainly involved in GO terms about the regulation of vasculature development, response to extracellular stimulus, and intrinsic apoptotic signaling pathway (Fig. 3C). GO Cluster plot showed the interaction between clusters and genes in GO terms (Fig. 3D). Relationships of different GO terms were visualized in Goplot (Fig. 3E). The down-regulated genes weren't enriched in any pathways.

**Fig. 4:**
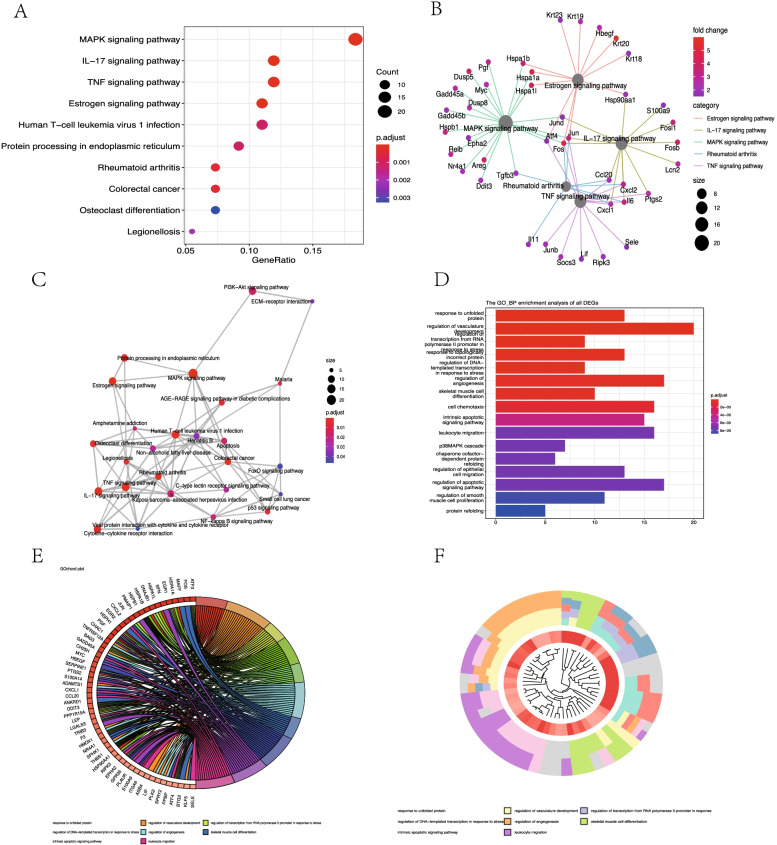
Gene Ontology (GO) and Kyoto Encyclopedia of Genes and Genomes analysis (KEGG) enrichment of up-regulated differentially expressed genes (DEGs) in GSE34351. **A** Advanced bubble chart shows significant KEGG pathways of the DEGs. **B** Cneplot visualized the conjunction between genes and the enrichment pathways. **C** Emaplot suggested the interaction of enriched pathways. **D** Bar chart visualized the GO enrichment significance items of DEGs. **E** Chord plot shows the distribution of DEGs in different GO terms. Gene symbols are presented on the left side of the graph and fold change values of DEGs are mapped by color scale. Gene involvement in the GO terms was determined by colored connecting lines. **F** GO Cluster plot showed the interaction between clusters and genes in GO terms

**Fig. 5:**
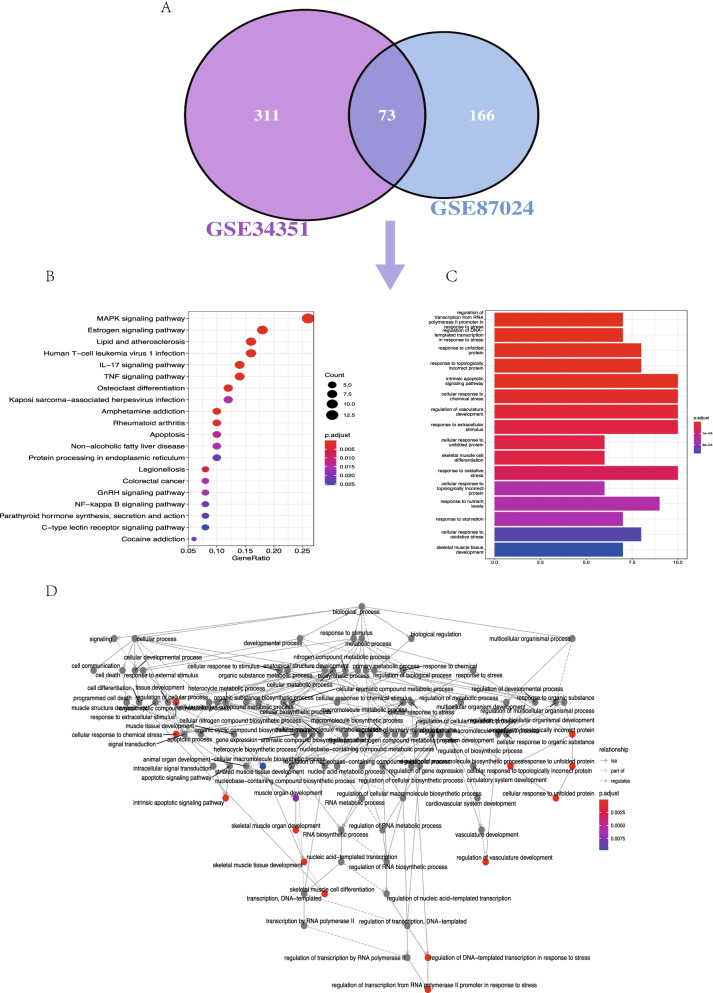
Visualization of the enrichment of common differentially expressed genes (DEGs) from two datasets. **A** Venn diagram presents a combination of all differentially expressed genes of two datasets. **B** Bar chart shows the significant KEGG pathways enriched by the 73 DEGs. **C** Bubble chart displays the significant GO terms. **D** GO plot shows the interacts with different enriched GO terms

For GSE34351, up-regulated genes were enriched in 28 significant pathways, including MAPK, IL-17, TNF, and Estrogen signaling pathways (Fig. 4A). Emaplot displayed the interaction of enriched pathways, and Cneplot visualized the interaction between genes and the enrichment pathways (Fig. 4B-C). Significant enrichment of GO terms included the unfolded protein, regulation of vasculature development, transcription from RNA polymerase II promoter to stress, and topologically incorrect protein (Fig. 4D). GO circle and cluster plot showed the distributions of the genes and GO terms (Fig. 4E-F). No pathway was enriched in the down-regulated genes. Venn diagram showed the common 73 DEGs from the two expressional datasets (Fig. 5A).

Combined DEGs enrichment analysis of GO and KEGG showed the significant pathways involved C − type lectin receptor, NF − kappa B, and GnRH signaling pathways (Table S1-2, Fig. 5B-D).

### GSEA of the two expression data sets

**Fig. 6:**
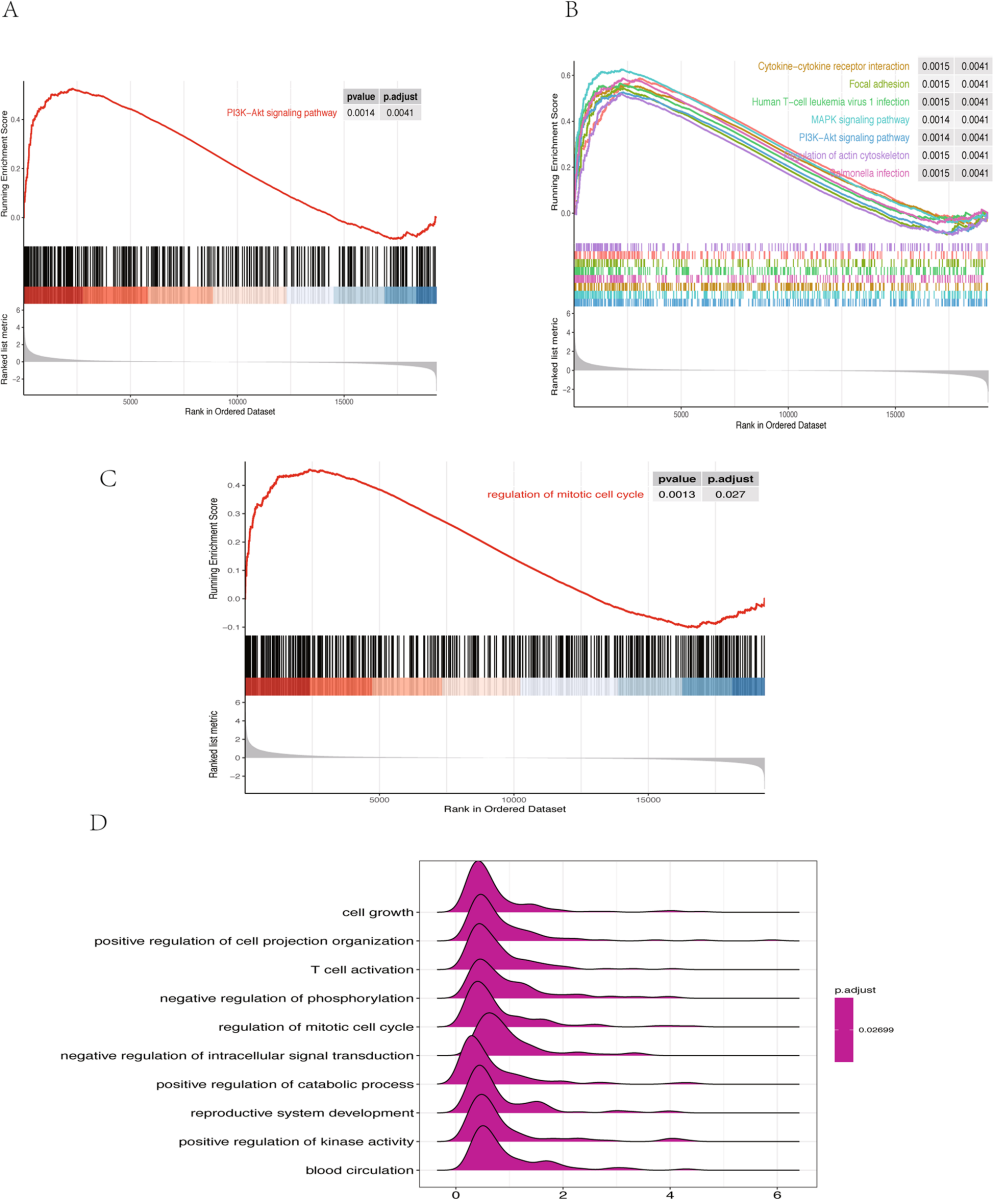
Visualization of the gene set enrichment analysis (GSEA) of the GSE87024. **A** GSEA plots shows the most enriched gene sets in KEGG of all detected genes in the GSE87024. **B** The top 10 most significant up-regulated enriched gene sets in KEGG. **E** The top terms enriched in GO database. **D** The top 10 most significantly enriched terms in GO database

**Fig. 7:**
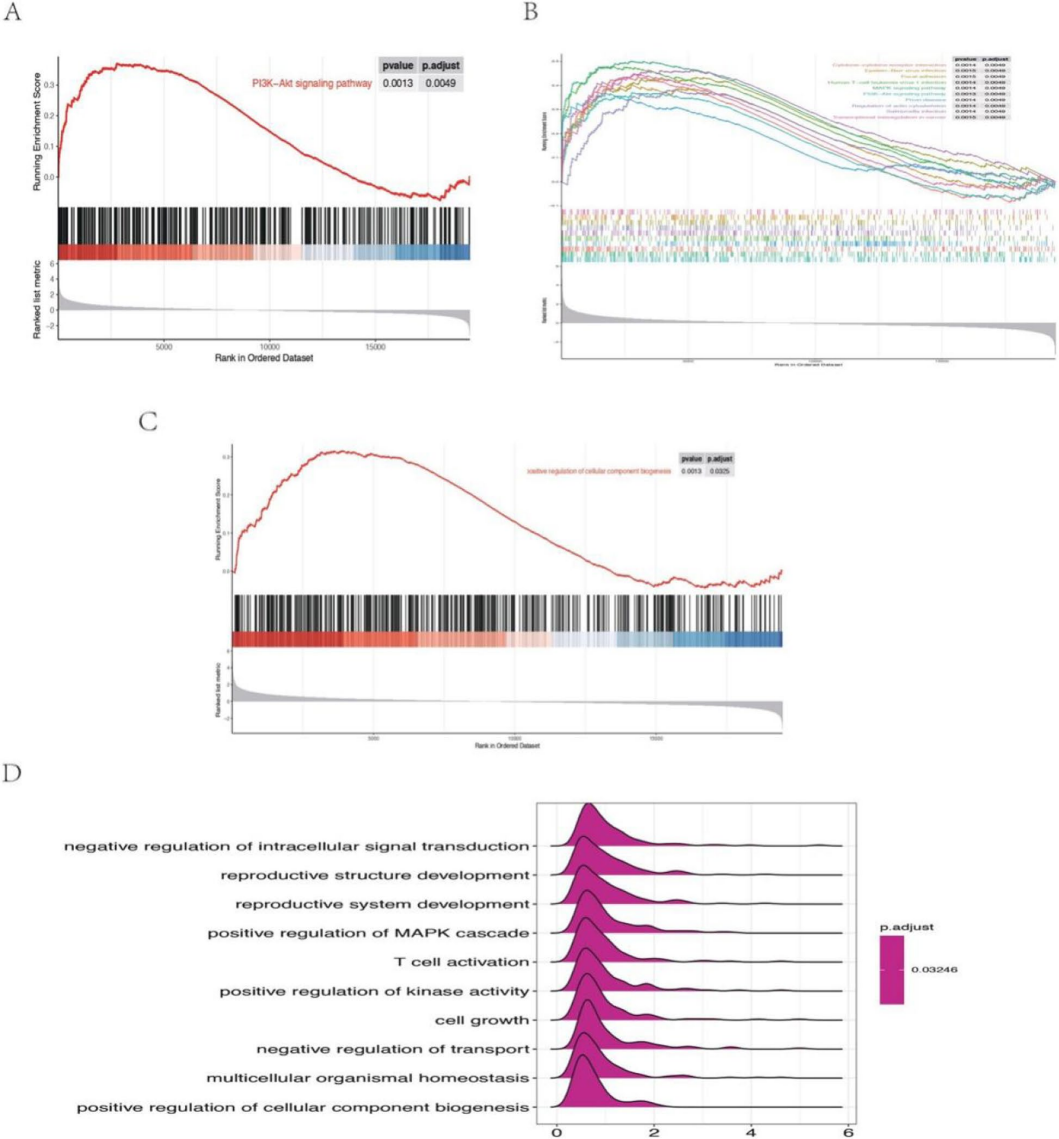
Visualization of the gene set enrichment analysis (GSEA) of the GSE34351. **A** GSEA plots shows the most enriched gene sets in KEGG of all detected genes in the GSE34351. **B** The top 10 most significant up-regulated enriched gene sets in KEGG. **E** The top terms enriched in GO database. **D** The top 10 most significantly enriched terms in GO database

GSEA of all detected genes in GSE87024 showed that the top KEGG gene set is the PI3K-Akt signaling pathway (Fig. 6A). The other possible mechanisms of IRI—AKI included MAPK signaling pathway and cytokine- cytokine receptor interaction in KEGG (Fig. 6B). The most significantly enriched gene set of GO terms was the mitotic cell cycle regulation (Fig. 6C). The other top 10 enrichment GO terms involved cell growth, upregulation of cell projection organization, T cell activation, and negative regulation of phosphorylation (Fig. 6D). For GSE34351, GSEA analysis of KEGG pathways was similar to the GSE87024 (Fig. 7A-B). GO terms indicated the biological process of the IRI-AKI development related to positive regulation of cellular component biogenesis, reproductive structure development, and positive regulation of MAPK cascade (Fig. 7C-D).

### Evaluation of immune cell infiltration

**Fig. 8:**
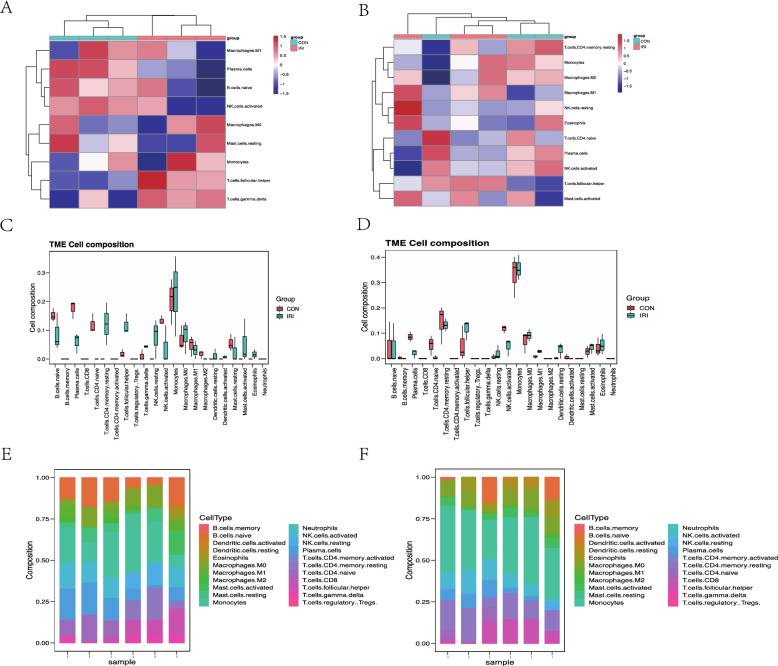
Evaluation and visualization of immune cell infiltration. **A** Heatmaps of the immune cell abundance in GSE87024; **B** Heatmaps of the immune cell abundance in GSE34351; **C** Barplot of the propotion of the immune cell in each sample (GSE87024). **D** Barplot of the propotion of the immune cell in each sample (GSE34351). **E** Box plot of the immune cell distribution in different group (GSE87024). **F** Box plot of the immune cell distribution in different group (GSE34351)

Immune cell infiltration analysis showed plasma cells, T cells CD4 naive decreased in IRI-AKI group, while T cells CD4 memory resting and T cells follicular helper increased in GSE87024. In GSE34351, macrophages M1 elevated while the plasma cells and NK cells reduced in IRI-AKI (Fig. 8).

### PPI network and analysis of hub genes and modules

**Fig. 9:**
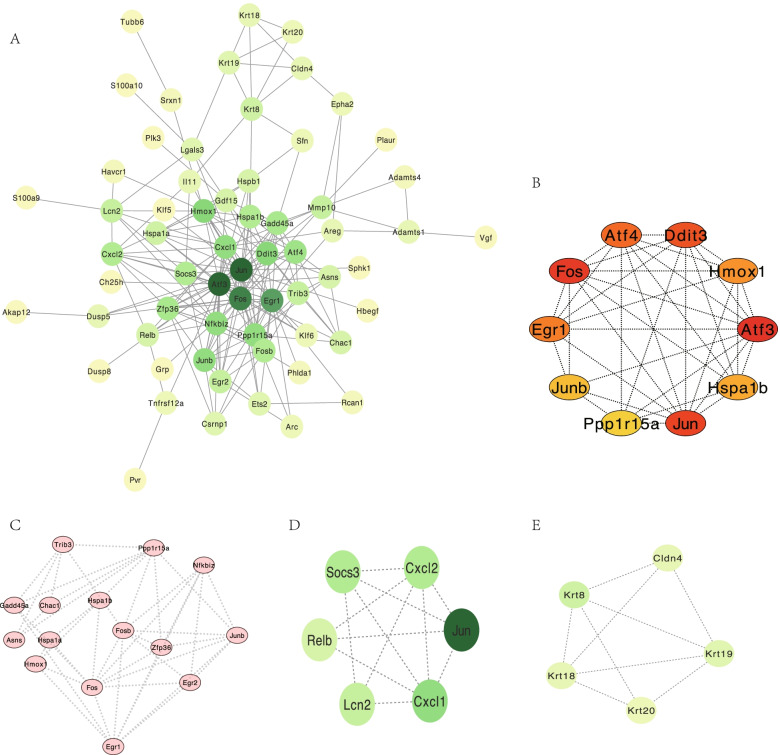
Outcomes of Protein–protein interaction (PPI) network. **A** PPI network of the common 73 differentially expressed genes (DEGs) of the 2 datasets. Node color reflects the grade of Degree scores calculated in the CytoNCA. (Green color represents a higher degree, and yellow color represents a lower degree). **B** 10 hub genes identified from the Cytohubba. **C**, **D**, **E** Subnetwork of hub modules acquired in the MCODE with MCC scores are 6, 4.8, and 4.5 respectively

The cluster of the PPI network of common 73 DEGs was composed of 73 nodes and 206 edges (Fig. 9A). The top 10 hub genes selected in the Cytohubba plug-in using the MCC method (score ≥ 5000) and node degree (score ≥ 10) included Activating Transcription Factor 3 (ATF3), FOS, JUN, DNA Damage Inducible Transcript 3 (DDIT3), Activating Transcription Factor 4 (ATF4), Early Growth Response 1 (EGR1), Heme Oxygenase 1 (HMOX1), Heat Shock Protein Family A Member 1B (HSPA1B), JUNB, and Protein Phosphatase 1 Regulatory Subunit 15A (PPP1R15A) (Fig. 9B). Applying CytoNCA, we obtained ten hub genes, namely JUN, ATF3, FOS, EGR1, HMOX1, DDIT3, JUNB, NF-kappa-B inhibitor zeta (NFKBIZ), PPP1R15A, and C-X-C Motif Chemokine Ligand 1 (CXCL1). Three hub modules were identified and the most significant module had 15 nodes (MCODE score = 6, Fig. 9C-E).

### RRA to find the combined DEGs

**Fig. 10:**
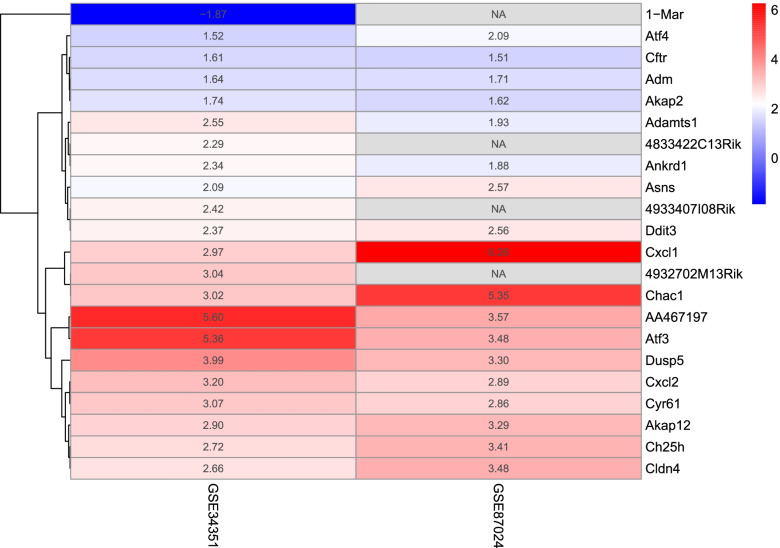
Heatmap of the Robust Rank Aggregated showed the top 20 differentially expressed genes aggregated of the two datasets. The red represents log FoldChange (FC) > 0, while green represents log FC < 0. The value in the box displays the log FC value

A total of 25 significant up-regulated genes and two down-regulated genes were identified in the RRA analysis. The heat map showed the expression profile of the top 20 most significant up and down-regulated genes. Each square represented a different gene, and each column represented the expression level of all genes in a data set (Fig. 10).

### TF-gene interactions and gene-miRNA network

**Fig. 11:**
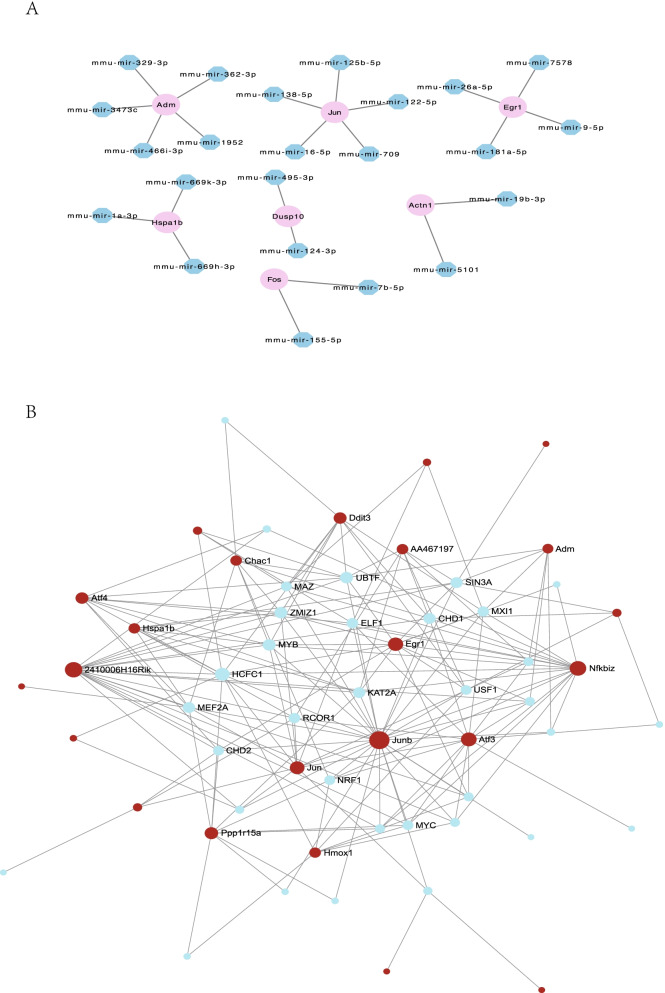
The networks of target gene-miRNA and TF-gene interactions. **A** Target gene-miRNA network. The pink circle nodes are the genes, and the blue octagon nodes are the miRNAs. **B** TF-gene interactions network. The red circle nodes are the genes, and blue octagon nodes are the transcription factors (TFs)

Gene-miRNA network showed both Adm and Jun modulated by 5 miRNAs, while Egr1 regulated by 3 miRNAs (Fig. 11A). The top 3 targeted DEGs for TFs were Junb, 2410006H16Rik, and Nfkbiz modulated by 26, 18, and 16 TFs separately (Fig. 11B).

### Construction of drug gene interaction network

**Fig. 12:**
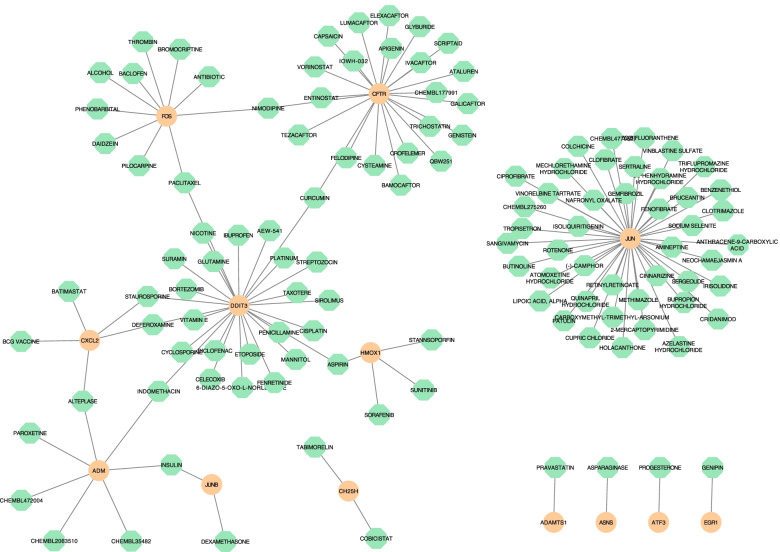
The drug-gene interaction network plot. The orange circle nodes are the genes, and the green octagon nodes are the drugs or molecular compounds

The drug-gene interaction network indicated that JUN, DDIT3, CFRT, FOS, ADM, interact with 44, 26, 22, 10, and 7 drugs or molecular compounds separately. The deferoxamine, glutamine, sirolimus, indomethacin are connected with JUN and DDIT3. (Table S3, Fig. 12).

## Discussion

In this study, we screened two expression profiles with the same tissue and similar genetic background mice in early-stage IRI-AKI from the GEO database, adopted RRA analysis to integrate the DEGs for detecting the potential biomarkers and pathways in the pathogenesis of IRI-AKI. We identified the MAPK, TNF, and IL-17 signaling pathways in KEGG database. Regulating the vasculature development, responding to extracellular stimulus, and intrinsic apoptotic signaling pathway were identified in GO database. Further GSEA analysis showed the PI3K-Akt signaling pathway, cytokine-cytokine receptor, positive regulation of cellular component biogenesis, and reproductive structure development pathway are crucial in IRI-AKI. Combined with the analysis by Cytohubba and CytoNCA, we figured out the JUN, ATF3, FOS, EGR1, HMOX1, DDIT3, JUNB, NFKBIZ, PPP1R15A, CXCL1, ATF4, and HSPA1B as hub genes.

GO, KEGG and GSEA analysis showed that the MAPK, TNF and IL-17 signaling pathways are crucial in IRI-AKI development. MAPK signaling pathway consists of four branches, namely ERK, JNK, p38, and ERK5. Activation of p38 and JNK signaling is a feature of acute kidney disease. The relative levels of JNK, p38, and ERK activation have been considered to determine cell fate after kidney damage. Selective inhibitors of p38 MAPK seemed to be effective in rodent models of acute kidney disease [20]. Remote ischemic pretreatment plays a role in preventing IRI from developing through activating JNK, p38, and MAPK kinase [21]. Several drugs or molecular compounds mitigate IRI via the MAPK pathway [22, 23]. TNF, considered as a crucial mediator in cell proliferation, cell death, and differentiation, interacts with two cell surface receptors: TNFR1 and TNFR2 (TNFRs) [24]. Studies showed the level of circulating TNF was increased during IRI-AKI causing renal cell damage via neutrophil-mediated inflammatory injury and apoptosis [25]. IRI mice with genetic deletion of TNFR1 displayed a significant lessening in renal injury and inflammation [26]. Pretreatment soluble TNFR2 fusion protein to neutralize TNF-a mitigate renal injury in IRI rats [27]. The IL-17 family consists of six members IL-17A-F and five members IL-17R A-E form the IL-17 receptor family. Researches showed IL-17A activation in IRI mice may promote inflammation activity. Administration of a neutralizing monoclonal anti-IL-17A antibody can attenuate renal damage by reducing pro-inflammatory mediators and enhancing renal and circulation levels of anti-inflammatory cytokines [28, 29]. Further researches are needed to detect the function of these pathways in IRI-AKI.

We identified 10 hub genes in IRI-AKI with one biomarker (Atf3) has been studied in IRI-AKI, five biomarkers (Cxcl1 and Jun, Fos, Nfkbiz, Hmox1) were researched in other types of AKI, 2 genes (Atf4 and Egr1) play role in I/R injury of other organs and three genes (Ppp1r15a, Hspa1b, and Ddit3) had not been reported in AKI or IRI researches. Atf3 could protect against IRI-AKI via suppressing p53 and inducing p21. In vitro studies showed it attenuated cell apoptosis by interacting with Nicotiflorin [30, 31]. CXCL1-CXCR2 signaling axis played an important role in alleviating cisplatin-induced AKI by regulation of inflammatory response [32]. Jun was studied in acute kidney injury including aristolochic acid-induced AKI, crush syndrome induced AKI, and myoglobinuric AKI but not IRI-AKI [33–35]. Inhibitor of c-Fos/activator protein-1 could decrease the production of TNF-a and other downstream molecules, which protect against LPS-AKI [36]. FosB induced the elevated expression of matrix metalloproteinase-2 in the cardiac IRI mice [37]. Studies found the NF-κB/miR-376b/NFKBIZ negative feedback loop adjusted intrarenal inflammation and alleviated renal damage in septic AKI [38]. HMOX1 long GT tandem repeats are associated with the occurrence of AKI in sickle cell anemia people [39]. Atf4 was related to endoplasmic reticulum stress, amino acid starvation, mitochondrial stress, and oxidative stress. It was reported that MIF-2/D-DT increased proximal tubular cell regeneration via ATF4-dependent pathways in IRI mice [40]. Egr1 was mainly studied in myocardial IRI and it may serve as a major regulator of remote preconditioning [41]. For Ppp1r15a, Hspa1b, and Ddit3, we haven't found any related AKI or IRI studies, which should be further verified in experimental studies.

In the gene-miRNA network analysis, mmu-mir-138-5p was found continuously increased in urine samples of rats daily administrated with gentamicin [42]. Researches showed that miR-709 was significantly upregulated in the proximal tubular cells of human and mice when suffering AKI [43]. However, there was no article about the roles of those miRNAs on IRI-AKI. Further studies were needed to examine the effects of these miRNAs on IRI-AKI.

We first conducted the drug-gene interaction network to identify the potential targets of IRI - AKI. Our results showed that staurosporine is a common molecular compounds interacting with CXCL2 and DDIT3. Considered as a protein kinase C inhibitor, staurosporine could protect against the impairment of working memory in IRI gerbils and rats [44, 45]. Curcumin interacts with both DDIT3 and cystic fibrosis transmembrane conductance regulator (CFTR). It is a diketone compound extracted from the plant turmeric. Some animal studies have shown that curcumin can protect the I/R injury and toxin-induced injury [46, 47]. Nowadays, researchers have designed a stepwise-targeting chitosan oligosaccharide conjugate, which can convey curcumin to renal tubular epithelial cells and remove excessive reactive oxygen species (ROS), to treat acute kidney injury [48]. Crofelemer, an inhibitor of the CFTR, was applied to alleviate pain in women with irritable bowel syndrome-diarrhea (IBS-D) as well as treat noninfectious diarrhea in HIV-positive patients receiving antiretroviral therapy [49]. Further studies should be conducted to discover the roles of the drugs or molecular compounds as potential therapeutic targets.

Our study have some strengths. First, we screened all the datasets about the IRI-AKI in GEO and focused on the early onset of this disease selecting the mice with similar genetic background to reduce individual differences. Second, we applied multiple bioinformatic methods to identify common DEGs that are potentially involved in the disease. In our limited knowledge, this was the first study applied the RRA analysis, a robust and compelling approach to integrate different datasets on IRI - AKI. Third, we performed the GSEA method utilizing all genetic expression information in datasets to find the crucial pathways in IRI-AKI. Different methods are applied to detect the hub genes and hub modules. Forth, we further analyzed the target genes for miRNA/TF. Fifth, we analyzed the signature of the immune cell in and found the T cell increasing in IRI-AKI. Last, we first conducted the drug-gene interaction network and identified 116 drugs or compounds as potential therapeutic targets of IRI-AKI giving new insights for further study.

There were some limitations in our study. First, to aggerate samples with similar genetic background mice and IRI-AKI occurrence time, we only selected two datasets and extracted a total of 12 samples. Though different times of IRI in GSE87024, we chose the earliest time after IRI-AKI to analyze. Since the limited sample numbers, we can't apply the weighted gene co-expression network analysis (WGCNA) to construct gene co-expression networks in our study. Second, we focused on the microarray and didn't include the RNA-seq, so we lack the data of miRNA and lncRNA. However, we constructed the TF-gene interactions and gene-miRNA network utilizing the open database. Third, the DEGs acquired from the RRA analysis are limited, we didn't perform further GO and KEGG pathway analysis. Fourth, we didn’t validate the hub genes identified in this study in AKI patients or experiment, which is a part of our future work.

## Conclusions

To conclude, our study identified 10 hub genes and 3 modules, key pathways involved in early IRI-AKI diagnosis and treatment utilizing various bioinformatic methods. We constructed the immune landscape and provided new insights and implications for further experimental confirmation.

## Supplementary Information

**Additional file 1:** **Table S1** KEGG enrichment outcomes of the common genes. **Table S2** GO analysis of the common genes. **Table S3** Drug gene interactions of the DEGs acquired from the RRA analysis and hub gens.

**Additional file 2:** **Figure S1** Box plots of the gene expression data after normalization.

## Data Availability

All data generated or analysed during this study are included in this published article and its Supplementary information files.

## Abbreviations

AKI: Acute kidney injury
ATF3: Activating Transcription Factor 3
ATF4: Activating Transcription Factor 4
CON: Control
CXCL1: C Motif Chemokine Ligand 1
DDIT3: DNA Damage Inducible Transcript 3
DEGs: Differentially expressed genes
DGIdb: Drug Gene Interaction Database
EGR1: Early Growth Response 1
GO: Gene ontology
GSEA: Gene set enrichment analysis
HMOX1: Heme Oxygenase 1
HSPA1B: Heat Shock Protein Family A Member 1B
IBS-D: Irritable bowel syndrome-diarrhea
IL-17: Interleukin-17
IL-18: Interleukin-18
IRI: Ischemia/reperfusion injury
KEGG: Kyoto Encyclopedia of Genes and Genomes
KIM-1: Kidney injury molecule-1
MAPK: Mitogen-activated protein kinase
MCODE: Molecular Complex Detection
NGAL: Neutrophil gelatinase-associated lipocalin
NFKBIZ: NF-kappa-B inhibitor zeta
PCA: Principal components analysis
PPI: Protein and protein interactions
PPP1R15A: Protein Phosphatase 1 Regulatory Subunit 15A
ROS: Reactive oxygen species
RRA: Robust rank aggregation
TF: Transcription factor
TNF: Tumor necrosis factor
WGCNA: Weighted gene co-expression network analysis
