# Supplementary material for: Identification of biomarkers, immune infiltration landscape, and treatment targets of ischemia–reperfusion acute kidney injury at an early stage by bioinformatics methods
Source: Hereditas. 2022 Jun 4;159:24. doi: 10.1186/s41065-022-00236-x (PMC9167514; doi:10.1186/s41065-022-00236-x)
Supplement: Supplementary file 1 — Additional file 1: Table S1 KEGG enrichment outcomes of the common genes. Table S2 GO analysis of the common genes. Table S3 Drug gene interactions of the DEGs acquired from the RRA analysis and hub gens. [file 41065_2022_236_MOESM1_ESM.docx]

TableS1. KEGG enrichment outcomes of the common genes.

| ID | Description | GeneRatio | BgRatio | pvalue | p.adjust | qvalue | geneID | Count |
| --- | --- | --- | --- | --- | --- | --- | --- | --- |
| mmu04010 | MAPK signaling pathway | 13/50 | 294/8918 | 4.95E-09 | 7.28E-07 | 5.89E-07 | Areg/Atf4/Ddit3/Dusp5/Dusp8/Epha2/Fos/Gadd45a/Hspa1a/Hspa1b/Hspb1/Jun/Relb | 13 |
| mmu04915 | Estrogen signaling pathway | 9/50 | 134/8918 | 4.43E-08 | 3.26E-06 | 2.64E-06 | Atf4/Fos/Hbegf/Hspa1a/Hspa1b/Jun/Krt18/Krt19/Krt20 | 9 |
| mmu04657 | IL-17signaling pathway | 7/50 | 91/8918 | 6.38E-07 | 3.13E-05 | 2.53E-05 | Cxcl1/Cxcl2/Fos/Fosb/Jun/Lcn2/S100a9 | 7 |
| mmu04668 | TNF signaling pathway | 7/50 | 113/8918 | 2.77E-06 | 0.000101873 | 8.24E-05 | Atf4/Cxcl1/Cxcl2/Fos/Jun/Junb/Socs3 | 7 |
| mmu05417 | Lipid and atherosclerosis | 8/50 | 216/8918 | 2.33E-05 | 0.000683985 | 0.000553457 | Atf4/Cxcl1/Cxcl2/Ddit3/Fos/Hspa1a/Hspa1b/Jun | 8 |
| mmu05031 | Amphetamine addiction | 5/50 | 69/8918 | 3.87E-05 | 0.000948857 | 0.000767783 | Arc/Atf4/Fos/Fosb/Jun | 5 |
| mmu05166 | Human T-cell leukemia virus 1 infection | 8/50 | 247/8918 | 6.06E-05 | 0.001273549 | 0.001030512 | Atf4/Egr1/Egr2/Ets2/Fos/Jun/Relb/Zfp36 | 8 |
| mmu04380 | Osteoclast differentiation | 6/50 | 128/8918 | 7.36E-05 | 0.001353149 | 0.001094922 | Fos/Fosb/Jun/Junb/Relb/Socs3 | 6 |
| mmu05323 | Rheumatoid arthritis | 5/50 | 87/8918 | 0.000118046 | 0.001928084 | 0.00156014 | Cxcl1/Cxcl2/Fos/Il11/Jun | 5 |
| mmu05134 | Legionellosis | 4/50 | 61/8918 | 0.000360669 | 0.005301841 | 0.004290068 | Cxcl1/Cxcl2/Hspa1a/Hspa1b | 4 |
| mmu04210 | Apoptosis | 5/50 | 136/8918 | 0.000935942 | 0.012507588 | 0.010120712 | Atf4/Ddit3/Fos/Gadd45a/Jun | 5 |
| mmu05210 | Colorectal cancer | 4/50 | 88/8918 | 0.001442299 | 0.015361379 | 0.012429902 | Areg/Fos/Gadd45a/Jun | 4 |
| mmu04932 | Non-alcoholic fatty liver disease | 5/50 | 151/8918 | 0.001493986 | 0.015361379 | 0.012429902 | Atf4/Ddit3/Fos/Jun/Socs3 | 5 |
| mmu05167 | Kaposi sarcoma-associated herpesvirus infection | 6/50 | 225/8918 | 0.001516123 | 0.015361379 | 0.012429902 | Cxcl1/Cxcl2/Fos/Jun/Rcan1/Zfp36 | 6 |
| mmu04912 | GnRH signaling pathway | 4/50 | 90/8918 | 0.001567488 | 0.015361379 | 0.012429902 | Atf4/Egr1/Hbegf/Jun | 4 |
| mmu05030 | Cocaine addiction | 3/50 | 48/8918 | 0.002402018 | 0.022068542 | 0.017857109 | Atf4/Fosb/Jun | 3 |
| mmu04141 | Protein processing in endoplasmic reticulum | 5/50 | 172/8918 | 0.002645147 | 0.022519076 | 0.018221665 | Atf4/Ddit3/Hspa1a/Hspa1b/Ppp1r15a | 5 |
| mmu04064 | NF-kappa B signaling pathway | 4/50 | 105/8918 | 0.002757438 | 0.022519076 | 0.018221665 | Cxcl1/Cxcl2/Gadd45a/Relb | 4 |
| mmu04928 | Parathyroid hormone synthesis, secretion and action | 4/50 | 108/8918 | 0.003053575 | 0.023625024 | 0.019116561 | Atf4/Egr1/Fos/Hbegf | 4 |
| mmu04625 | C-type lectin receptor signaling pathway | 4/50 | 112/8918 | 0.003481359 | 0.025534675 | 0.020661785 | Egr2/Jun/Plk3/Relb | 4 |
| mmu05020 | Prion disease | 6/50 | 268/8918 | 0.003647811 | 0.025534675 | 0.020661785 | Atf4/Ddit3/Egr1/Hspa1a/Hspa1b/Tubb6 | 6 |
| mmu04935 | Growth hormone synthesis, secretion and action | 4/50 | 116/8918 | 0.00394833 | 0.026382026 | 0.021347433 | Atf4/Fos/Junb/Socs3 | 4 |

Table S2. GO analysis of the common genes

| ID | Description | GeneRatio | BgRatio | pvalue | p.adjust | qvalue | Count |
| --- | --- | --- | --- | --- | --- | --- | --- |
| GO:0043618 | regulation of transcription from RNA polymerase II promoter in response to stress | 7/72 | 46/23328 | 9.62E-11 | 1.71E-07 | 1.22E-07 | 7 |
| GO:0043620 | regulation of DNA-templated transcription in response to stress | 7/72 | 51/23328 | 2.06E-10 | 1.83E-07 | 1.30E-07 | 7 |
| GO:0006986 | response to unfolded protein | 8/72 | 117/23328 | 2.88E-09 | 1.70E-06 | 1.22E-06 | 8 |
| GO:0035966 | response to topologically incorrect protein | 8/72 | 141/23328 | 1.26E-08 | 5.60E-06 | 4.00E-06 | 8 |
| GO:0097193 | intrinsic apoptotic signaling pathway | 10/72 | 295/23328 | 2.41E-08 | 8.57E-06 | 6.12E-06 | 10 |
| GO:0062197 | cellular response to chemical stress | 10/72 | 332/23328 | 7.31E-08 | 2.17E-05 | 1.55E-05 | 10 |
| GO:1901342 | regulation of vasculature development | 10/72 | 356/23328 | 1.40E-07 | 3.55E-05 | 2.53E-05 | 10 |
| GO:0009991 | response to extracellular stimulus | 10/72 | 378/23328 | 2.43E-07 | 5.40E-05 | 3.86E-05 | 10 |
| GO:0034620 | cellular response to unfolded protein | 6/72 | 88/23328 | 3.10E-07 | 5.51E-05 | 3.93E-05 | 6 |
| GO:0035914 | skeletal muscle cell differentiation | 6/72 | 88/23328 | 3.10E-07 | 5.51E-05 | 3.93E-05 | 6 |
| GO:0006979 | response to oxidative stress | 10/72 | 409/23328 | 5.01E-07 | 8.09E-05 | 5.77E-05 | 10 |
| GO:0035967 | cellular response to topologically incorrect protein | 6/72 | 110/23328 | 1.16E-06 | 0.00016016 | 0.000114322 | 6 |
| GO:0031667 | response to nutrient levels | 9/72 | 346/23328 | 1.17E-06 | 0.00016016 | 0.000114322 | 9 |
| GO:0042594 | response to starvation | 7/72 | 178/23328 | 1.30E-06 | 0.000164742 | 0.000117593 | 7 |
| GO:0034599 | cellular response to oxidative stress | 8/72 | 276/23328 | 2.16E-06 | 0.000255525 | 0.000182394 | 8 |
| GO:0007519 | skeletal muscle tissue development | 7/72 | 196/23328 | 2.47E-06 | 0.000273781 | 0.000195425 | 7 |
| GO:0060538 | skeletal muscle organ development | 7/72 | 206/23328 | 3.43E-06 | 0.000358148 | 0.000255646 | 7 |
| GO:0060326 | cell chemotaxis | 8/72 | 303/23328 | 4.30E-06 | 0.000424453 | 0.000302974 | 8 |
| GO:1990440 | positive regulation of transcription from RNA polymerase II promoter in response to endoplasmic reticulum stress | 3/72 | 11/23328 | 4.57E-06 | 0.000427382 | 0.000305065 | 3 |
| GO:0001933 | negative regulation of protein phosphorylation | 9/72 | 425/23328 | 6.27E-06 | 0.000542807 | 0.000387455 | 9 |
| GO:0045765 | regulation of angiogenesis | 8/72 | 320/23328 | 6.41E-06 | 0.000542807 | 0.000387455 | 8 |
| GO:0097191 | extrinsic apoptotic signaling pathway | 7/72 | 233/23328 | 7.69E-06 | 0.000603315 | 0.000430645 | 7 |
| GO:0032436 | positive regulation of proteasomal ubiquitin-dependent protein catabolic process | 5/72 | 88/23328 | 7.81E-06 | 0.000603315 | 0.000430645 | 5 |
| GO:1902895 | positive regulation of pri-miRNA transcription by RNA polymerase II | 4/72 | 44/23328 | 1.03E-05 | 0.000763561 | 0.000545029 | 4 |
| GO:0034976 | response to endoplasmic reticulum stress | 7/72 | 249/23328 | 1.18E-05 | 0.000841637 | 0.00060076 | 7 |
| GO:0042326 | negative regulation of phosphorylation | 9/72 | 471/23328 | 1.42E-05 | 0.00096383 | 0.000687981 | 9 |
| GO:0038066 | p38MAPK cascade | 4/72 | 48/23328 | 1.46E-05 | 0.00096383 | 0.000687981 | 4 |
| GO:2000060 | positive regulation of ubiquitin-dependent protein catabolic process | 5/72 | 101/23328 | 1.53E-05 | 0.000971356 | 0.000693353 | 5 |
| GO:0048661 | positive regulation of smooth muscle cell proliferation | 5/72 | 108/23328 | 2.12E-05 | 0.001297951 | 0.000926476 | 5 |
| GO:1901800 | positive regulation of proteasomal protein catabolic process | 5/72 | 109/23328 | 2.21E-05 | 0.00131187 | 0.000936412 | 5 |
| GO:0071496 | cellular response to external stimulus | 7/72 | 280/23328 | 2.52E-05 | 0.001444421 | 0.001031026 | 7 |
| GO:0035994 | response to muscle stretch | 3/72 | 19/23328 | 2.64E-05 | 0.001461076 | 0.001042915 | 3 |
| GO:1902893 | regulation of pri-miRNA transcription by RNA polymerase II | 4/72 | 56/23328 | 2.71E-05 | 0.001461076 | 0.001042915 | 4 |
| GO:0031331 | positive regulation of cellular catabolic process | 8/72 | 396/23328 | 2.98E-05 | 0.001557343 | 0.00111163 | 8 |
| GO:0061614 | pri-miRNA transcription by RNA polymerase II | 4/72 | 58/23328 | 3.12E-05 | 0.00158391 | 0.001130593 | 4 |
| GO:0000302 | response to reactive oxygen species | 6/72 | 202/23328 | 3.80E-05 | 0.001876413 | 0.001339382 | 6 |
| GO:2001233 | regulation of apoptotic signaling pathway | 8/72 | 413/23328 | 4.01E-05 | 0.001925743 | 0.001374594 | 8 |
| GO:1903052 | positive regulation of proteolysis involved in cellular protein catabolic process | 5/72 | 124/23328 | 4.12E-05 | 0.001925743 | 0.001374594 | 5 |
| GO:1901216 | positive regulation of neuron death | 5/72 | 126/23328 | 4.45E-05 | 0.00202068 | 0.00144236 | 5 |
| GO:0070059 | intrinsic apoptotic signaling pathway in response to endoplasmic reticulum stress | 4/72 | 64/23328 | 4.61E-05 | 0.00202068 | 0.00144236 | 4 |
| GO:0036003 | positive regulation of transcription from RNA polymerase II promoter in response to stress | 3/72 | 23/23328 | 4.78E-05 | 0.00202068 | 0.00144236 | 3 |
| GO:0071276 | cellular response to cadmium ion | 3/72 | 23/23328 | 4.78E-05 | 0.00202068 | 0.00144236 | 3 |
| GO:0032434 | regulation of proteasomal ubiquitin-dependent protein catabolic process | 5/72 | 131/23328 | 5.35E-05 | 0.002212191 | 0.00157906 | 5 |
| GO:0031668 | cellular response to extracellular stimulus | 6/72 | 217/23328 | 5.66E-05 | 0.002287331 | 0.001632695 | 6 |
| GO:0030968 | endoplasmic reticulum unfolded protein response | 4/72 | 68/23328 | 5.85E-05 | 0.002310309 | 0.001649096 | 4 |
| GO:1903364 | positive regulation of cellular protein catabolic process | 5/72 | 145/23328 | 8.67E-05 | 0.003236281 | 0.002310054 | 5 |
| GO:1900745 | positive regulation of p38MAPK cascade | 3/72 | 28/23328 | 8.74E-05 | 0.003236281 | 0.002310054 | 3 |
| GO:0010632 | regulation of epithelial cell migration | 6/72 | 235/23328 | 8.79E-05 | 0.003236281 | 0.002310054 | 6 |
| GO:0009896 | positive regulation of catabolic process | 8/72 | 463/23328 | 8.92E-05 | 0.003236281 | 0.002310054 | 8 |
| GO:0045862 | positive regulation of proteolysis | 7/72 | 344/23328 | 9.24E-05 | 0.003282862 | 0.002343304 | 7 |
| GO:0071277 | cellular response to calcium ion | 4/72 | 77/23328 | 9.52E-05 | 0.003317274 | 0.002367867 | 4 |
| GO:0043525 | positive regulation of neuron apoptotic process | 4/72 | 81/23328 | 0.000115965 | 0.003951164 | 0.002820338 | 4 |
| GO:0045742 | positive regulation of epidermal growth factor receptor signaling pathway | 3/72 | 31/23328 | 0.00011909 | 0.003951164 | 0.002820338 | 3 |
| GO:0009267 | cellular response to starvation | 5/72 | 156/23328 | 0.000122293 | 0.003951164 | 0.002820338 | 5 |
| GO:0071248 | cellular response to metal ion | 5/72 | 156/23328 | 0.000122293 | 0.003951164 | 0.002820338 | 5 |
| GO:2000058 | regulation of ubiquitin-dependent protein catabolic process | 5/72 | 159/23328 | 0.000133719 | 0.004228488 | 0.003018291 | 5 |
| GO:0009611 | response to wounding | 8/72 | 492/23328 | 0.000135635 | 0.004228488 | 0.003018291 | 8 |
| GO:0019221 | cytokine-mediated signaling pathway | 7/72 | 369/23328 | 0.000142614 | 0.004262187 | 0.003042345 | 7 |
| GO:0060706 | cell differentiation involved in embryonic placenta development | 3/72 | 33/23328 | 0.000143912 | 0.004262187 | 0.003042345 | 3 |
| GO:1901186 | positive regulation of ERBB signaling pathway | 3/72 | 33/23328 | 0.000143912 | 0.004262187 | 0.003042345 | 3 |
| GO:0051348 | negative regulation of transferase activity | 6/72 | 260/23328 | 0.000152772 | 0.004450414 | 0.003176701 | 6 |
| GO:0048660 | regulation of smooth muscle cell proliferation | 5/72 | 165/23328 | 0.000159011 | 0.004557466 | 0.003253115 | 5 |
| GO:0046686 | response to cadmium ion | 3/72 | 35/23328 | 0.000171874 | 0.004847927 | 0.003460446 | 3 |
| GO:0071356 | cellular response to tumor necrosis factor | 5/72 | 169/23328 | 0.000177802 | 0.004936777 | 0.003523867 | 5 |
| GO:0048659 | smooth muscle cell proliferation | 5/72 | 170/23328 | 0.000182754 | 0.004996205 | 0.003566287 | 5 |
| GO:2000273 | positive regulation of signaling receptor activity | 3/72 | 37/23328 | 0.000203141 | 0.005469429 | 0.003904074 | 3 |
| GO:0043535 | regulation of blood vessel endothelial cell migration | 4/72 | 94/23328 | 0.000206232 | 0.005469773 | 0.00390432 | 4 |
| GO:0030593 | neutrophil chemotaxis | 4/72 | 99/23328 | 0.000251641 | 0.006575959 | 0.004693914 | 4 |
| GO:2001235 | positive regulation of apoptotic signaling pathway | 5/72 | 184/23328 | 0.000263757 | 0.006792707 | 0.004848629 | 5 |
| GO:1900744 | regulation of p38MAPK cascade | 3/72 | 41/23328 | 0.000276244 | 0.007012639 | 0.005005616 | 3 |
| GO:0031669 | cellular response to nutrient levels | 5/72 | 188/23328 | 0.000291258 | 0.007145505 | 0.005100456 | 5 |
| GO:0034612 | response to tumor necrosis factor | 5/72 | 188/23328 | 0.000291258 | 0.007145505 | 0.005100456 | 5 |
| GO:0070997 | neuron death | 7/72 | 416/23328 | 0.000296287 | 0.007145505 | 0.005100456 | 7 |
| GO:0061136 | regulation of proteasomal protein catabolic process | 5/72 | 189/23328 | 0.000298462 | 0.007145505 | 0.005100456 | 5 |
| GO:0010631 | epithelial cell migration | 6/72 | 295/23328 | 0.000301583 | 0.007145505 | 0.005100456 | 6 |
| GO:0090132 | epithelium migration | 6/72 | 297/23328 | 0.000312655 | 0.007244024 | 0.005170778 | 6 |
| GO:0007517 | muscle organ development | 7/72 | 420/23328 | 0.000313894 | 0.007244024 | 0.005170778 | 7 |
| GO:0090130 | tissue migration | 6/72 | 299/23328 | 0.000324044 | 0.007382385 | 0.00526954 | 6 |
| GO:0001667 | ameboidal-type cell migration | 7/72 | 427/23328 | 0.000346726 | 0.007799151 | 0.005567027 | 7 |
| GO:0043491 | protein kinase B signaling | 5/72 | 196/23328 | 0.000352741 | 0.007835264 | 0.005592804 | 5 |
| GO:0001892 | embryonic placenta development | 4/72 | 114/23328 | 0.000430814 | 0.009451316 | 0.006746341 | 4 |
| GO:0030216 | keratinocyte differentiation | 4/72 | 115/23328 | 0.000445305 | 0.009646313 | 0.00688553 | 4 |
| GO:0006469 | negative regulation of protein kinase activity | 5/72 | 207/23328 | 0.000452746 | 0.009646313 | 0.00688553 | 5 |
| GO:0014706 | striated muscle tissue development | 7/72 | 447/23328 | 0.000455988 | 0.009646313 | 0.00688553 | 7 |
| GO:1904018 | positive regulation of vasculature development | 5/72 | 208/23328 | 0.000462795 | 0.009675129 | 0.006906099 | 5 |
| GO:1902041 | regulation of extrinsic apoptotic signaling pathway via death domain receptors | 3/72 | 49/23328 | 0.000469072 | 0.009692326 | 0.006918374 | 3 |

TableS3. Drug gene interactions of the DEGs acquired from the RRA analysis and hub genes.

| Search_term | Match_term | Match_type | Gene | Drug | Interaction_types | Sources | Pmids |
| --- | --- | --- | --- | --- | --- | --- | --- |
| JUN | JUN | Definite | JUN | BUPROPION HYDROCHLORIDE |  | DTC |  |
| JUN | JUN | Definite | JUN | BUTINOLINE |  | DTC |  |
| JUN | JUN | Definite | JUN | CLOTRIMAZOLE |  | DTC | 16680159 |
| JUN | JUN | Definite | JUN | BENZENETHIOL |  | DTC |  |
| JUN | JUN | Definite | JUN | CARBOXYMETHYL-TRIMETHYL-ARSONIUM |  | DTC |  |
| JUN | JUN | Definite | JUN | CHEMBL275260 |  | DTC |  |
| JUN | JUN | Definite | JUN | FENOFIBRATE |  | DTC | 16680159 |
| JUN | JUN | Definite | JUN | MECHLORETHAMINE HYDROCHLORIDE |  | DTC |  |
| JUN | JUN | Definite | JUN | SODIUM SELENITE |  | DTC |  |
| JUN | JUN | Definite | JUN | VINORELBINE TARTRATE |  | DTC |  |
| JUN | JUN | Definite | JUN | TROPISETRON |  | DTC |  |
| JUN | JUN | Definite | JUN | CINNARIZINE |  | DTC | 16680159 |
| JUN | JUN | Definite | JUN | VINBLASTINE SULFATE |  | DTC |  |
| JUN | JUN | Definite | JUN | CUPRIC CHLORIDE |  | DTC |  |
| JUN | JUN | Definite | JUN | TRIFLUPROMAZINE HYDROCHLORIDE |  | DTC |  |
| JUN | JUN | Definite | JUN | COLCHICINE |  | DTC |  |
| JUN | JUN | Definite | JUN | CIPROFIBRATE |  | DTC | 16680159 |
| JUN | JUN | Definite | JUN | NAFRONYL OXALATE |  | DTC |  |
| JUN | JUN | Definite | JUN | BRUCEANTIN |  | DTC | 19199792 |
| JUN | JUN | Definite | JUN | METHIMAZOLE |  | DTC |  |
| JUN | JUN | Definite | JUN | SERGEOLIDE |  | DTC | 19199792 |
| JUN | JUN | Definite | JUN | DIPHENHYDRAMINE HYDROCHLORIDE |  | DTC |  |
| JUN | JUN | Definite | JUN | IRISOLIDONE |  | DTC | 17996449 |
| JUN | JUN | Definite | JUN | ROTENONE |  | DTC | 16680159 |
| JUN | JUN | Definite | JUN | CRIDANIMOD |  | DTC |  |
| JUN | JUN | Definite | JUN | BENZO[B]FLUORANTHENE |  | DTC |  |
| JUN | JUN | Definite | JUN | 2-MERCAPTOPYRIMIDINE |  | DTC |  |
| JUN | JUN | Definite | JUN | SANGIVAMYCIN |  | DTC | 17371872 |
| JUN | JUN | Definite | JUN | ANTHRACENE-9-CARBOXYLIC ACID |  | DTC |  |
| JUN | JUN | Definite | JUN | AMINEPTINE |  | DTC |  |
| JUN | JUN | Definite | JUN | AZELASTINE HYDROCHLORIDE |  | DTC |  |
| JUN | JUN | Definite | JUN | PATULIN |  | DTC | 16680159 |
| JUN | JUN | Definite | JUN | CLOFIBRATE |  | DTC | 16680159 |
| JUN | JUN | Definite | JUN | QUINAPRIL HYDROCHLORIDE |  | DTC |  |
| JUN | JUN | Definite | JUN | RETINYLRETINOATE |  | DTC | 18511283 |
| JUN | JUN | Definite | JUN | LIPOIC ACID, ALPHA |  | DTC |  |
| JUN | JUN | Definite | JUN | ISOLIQUIRITIGENIN |  | DTC | 21866899 |
| JUN | JUN | Definite | JUN | ATOMOXETINE HYDROCHLORIDE |  | DTC |  |
| JUN | JUN | Definite | JUN | NEOCHAMAEJASMIN A |  | DTC | 18380477 |
| JUN | JUN | Definite | JUN | GEMFIBROZIL |  | DTC | 16680159 |
| JUN | JUN | Definite | JUN | SERTRALINE |  | DTC | 16680159 |
| JUN | JUN | Definite | JUN | (-)-CAMPHOR |  | DTC |  |
| JUN | JUN | Definite | JUN | CHEMBL477052 |  | DTC | 21504179 |
| JUN | JUN | Definite | JUN | HOLACANTHONE |  | DTC | 19199792 |
| FOS | FOS | Definite | FOS | DAIDZEIN |  | NCI | 8593857 |
| FOS | FOS | Definite | FOS | PHENOBARBITAL |  | NCI | 9187311 |
| FOS | FOS | Definite | FOS | THROMBIN |  | NCI | 2119237 |
| FOS | FOS | Definite | FOS | NIMODIPINE |  | NCI | 8301097 |
| FOS | FOS | Definite | FOS | ANTIBIOTIC |  | NCI | 15773551 |
| FOS | FOS | Definite | FOS | ALCOHOL |  | NCI | 16750178 |
| FOS | FOS | Definite | FOS | PACLITAXEL |  | NCI | 9588740 |
| FOS | FOS | Definite | FOS | PILOCARPINE |  | NCI | 9661997 |
| FOS | FOS | Definite | FOS | BACLOFEN |  | NCI | 11301212 |
| FOS | FOS | Definite | FOS | BROMOCRIPTINE |  | NCI | 11680511 |
| EGR1 | EGR1 | Definite | EGR1 | GENIPIN |  | DTC | 26283511 |
| HMOX1 | HMOX1 | Definite | HMOX1 | SORAFENIB |  | CIViC | 26309414 |
| HMOX1 | HMOX1 | Definite | HMOX1 | SUNITINIB |  | CIViC | 26309414 |
| HMOX1 | HMOX1 | Definite | HMOX1 | STANNSOPORFIN |  | TdgClinicalTrial\|TTD | 12785007\|15686268\|8321837\|8265722\|10872746 |
| HMOX1 | HMOX1 | Definite | HMOX1 | ASPIRIN |  | NCI | 12927812 |
| DDIT3 | DDIT3 | Definite | DDIT3 | 6-DIAZO-5-OXO-L-NORLEUCINE |  | NCI | 10377266 |
| DDIT3 | DDIT3 | Definite | DDIT3 | DEFEROXAMINE |  | NCI | 8794898 |
| DDIT3 | DDIT3 | Definite | DDIT3 | VITAMIN E |  | NCI | 15271854 |
| DDIT3 | DDIT3 | Definite | DDIT3 | TAXOTERE |  | NCI | 10470115 |
| DDIT3 | DDIT3 | Definite | DDIT3 | STAUROSPORINE |  | NCI | 17167033 |
| DDIT3 | DDIT3 | Definite | DDIT3 | ASPIRIN |  | NCI | 10688535 |
| DDIT3 | DDIT3 | Definite | DDIT3 | INDOMETHACIN |  | NCI | 15131590 |
| DDIT3 | DDIT3 | Definite | DDIT3 | SIROLIMUS |  | NCI | 11145585 |
| DDIT3 | DDIT3 | Definite | DDIT3 | CISPLATIN |  | NCI | 10071988\|8554977 |
| DDIT3 | DDIT3 | Definite | DDIT3 | IBUPROFEN |  | NCI | 15131590 |
| DDIT3 | DDIT3 | Definite | DDIT3 | SURAMIN |  | NCI | 9586958 |
| DDIT3 | DDIT3 | Definite | DDIT3 | PENICILLAMINE |  | NCI | 11526215 |
| DDIT3 | DDIT3 | Definite | DDIT3 | PLATINUM |  | NCI | 8996528 |
| DDIT3 | DDIT3 | Definite | DDIT3 | GLUTAMINE |  | NCI | 10377266 |
| DDIT3 | DDIT3 | Definite | DDIT3 | DICLOFENAC |  | NCI | 15131590 |
| DDIT3 | DDIT3 | Definite | DDIT3 | PACLITAXEL |  | NCI | 8554977 |
| DDIT3 | DDIT3 | Definite | DDIT3 | FENRETINIDE |  | NCI | 12234979\|17273769 |
| DDIT3 | DDIT3 | Definite | DDIT3 | MANNITOL |  | NCI | 8670069 |
| DDIT3 | DDIT3 | Definite | DDIT3 | BORTEZOMIB |  | NCI | 16357160 |
| DDIT3 | DDIT3 | Definite | DDIT3 | ETOPOSIDE |  | NCI | 9044846 |
| DDIT3 | DDIT3 | Definite | DDIT3 | CURCUMIN |  | NCI | 15271854 |
| DDIT3 | DDIT3 | Definite | DDIT3 | STREPTOZOCIN |  | NCI | 8482431 |
| DDIT3 | DDIT3 | Definite | DDIT3 | NICOTINE |  | NCI | 11592233 |
| DDIT3 | DDIT3 | Definite | DDIT3 | CELECOXIB |  | NCI\|PharmGKB | 17167033\|17166886\|15131590\|16597647\|22336956 |
| DDIT3 | DDIT3 | Definite | DDIT3 | AEW-541 |  | CIViC | 28637688 |
| DDIT3 | DDIT3 | Definite | DDIT3 | CYCLOSPORINE |  | NCI | 15494209 |
| JUNB | JUNB | Definite | JUNB | INSULIN |  | NCI | 8264634 |
| JUNB | JUNB | Definite | JUNB | DEXAMETHASONE |  | NCI | 9024781 |
| ADAMTS1 | ADAMTS1 | Definite | ADAMTS1 | PRAVASTATIN |  | PharmGKB | 18174457 |
| ADM | ADM | Definite | ADM | CHEMBL472004 |  | DTC | 22884224 |
| ADM | ADM | Definite | ADM | INSULIN |  | NCI | 12668590 |
| ADM | ADM | Definite | ADM | CHEMBL35482 |  | NCI | 10830296 |
| ADM | ADM | Definite | ADM | PAROXETINE |  | PharmGKB | 19636336 |
| ADM | ADM | Definite | ADM | ALTEPLASE |  | NCI | 10848972 |
| ADM | ADM | Definite | ADM | CHEMBL2063510 |  | DTC | 22884224 |
| ADM | ADM | Definite | ADM | INDOMETHACIN |  | NCI | 10830296 |
| ASNS | ASNS | Definite | ASNS | ASPARAGINASE |  | NCI\|CIViC\|PharmGKB | 28069604\|24268318\|11556848 |
| ATF3 | ATF3 | Definite | ATF3 | PROGESTERONE |  | NCI | 15674352 |
| CFTR | CFTR | Definite | CFTR | NIMODIPINE | activator | GuideToPharmacology |  |
| CFTR | CFTR | Definite | CFTR | APIGENIN | activator | GuideToPharmacology |  |
| CFTR | CFTR | Definite | CFTR | CROFELEMER | inhibitor\|antagonist | TdgClinicalTrial\|ChemblInteractions\|GuideToPharmacology\|TTD | 19808995 |
| CFTR | CFTR | Definite | CFTR | QBW251 | activator | ChemblInteractions |  |
| CFTR | CFTR | Definite | CFTR | FELODIPINE | activator | GuideToPharmacology |  |
| CFTR | CFTR | Definite | CFTR | CAPSAICIN | activator | GuideToPharmacology |  |
| CFTR | CFTR | Definite | CFTR | LUMACAFTOR | modulator | DTC\|TdgClinicalTrial\|ChemblInteractions\|GuideToPharmacology\|PharmGKB\|TTD\|FDA | 26041577\|22698459\|24561283\|26137539\|24038832\|22293084\|26416827\|24973281\|21825083\|24392786\|26823392\|28325531\|24796242\|27334259\|21976485\|23891399 |
| CFTR | CFTR | Definite | CFTR | CHEMBL177991 | activator | GuideToPharmacology |  |
| CFTR | CFTR | Definite | CFTR | GENISTEIN | activator | NCI\|GuideToPharmacology\|PharmGKB | 27261451\|12124395 |
| CFTR | CFTR | Definite | CFTR | IVACAFTOR | activator\|potentiator | TdgClinicalTrial\|ChemblInteractions\|GuideToPharmacology\|TTD\|FDA | 27636560\|22293084 |
| CFTR | CFTR | Definite | CFTR | GLYBURIDE | blocker\|antagonist | GuideToPharmacology | 12407077\|12202948\|12391048\|14729151\|15365090 |
| CFTR | CFTR | Definite | CFTR | ELEXACAFTOR | modulator | GuideToPharmacology\|FDA |  |
| CFTR | CFTR | Definite | CFTR | TEZACAFTOR | activator | ChemblInteractions\|GuideToPharmacology\|PharmGKB\|TTD\|FDA | 28930490\|27714410\|29099333\|29099344 |
| CFTR | CFTR | Definite | CFTR | SCRIPTAID |  | DTC | 19966789 |
| CFTR | CFTR | Definite | CFTR | VORINOSTAT |  | DTC | 19966789 |
| CFTR | CFTR | Definite | CFTR | ENTINOSTAT |  | DTC | 19966789 |
| CFTR | CFTR | Definite | CFTR | CYSTEAMINE |  | PharmGKB | 27035618 |
| CFTR | CFTR | Definite | CFTR | ATALUREN |  | PharmGKB | 18722008\|21233271\|26840186\|20622033 |
| CFTR | CFTR | Definite | CFTR | TRICHOSTATIN |  | DTC | 19966789 |
| CFTR | CFTR | Definite | CFTR | GALICAFTOR |  | TTD |  |
| CFTR | CFTR | Definite | CFTR | BAMOCAFTOR |  | GuideToPharmacology |  |
| CFTR | CFTR | Definite | CFTR | IOWH-032 |  | TTD |  |
| CFTR | CFTR | Definite | CFTR | CURCUMIN |  | PharmGKB | 27007499 |
| CH25H | CH25H | Definite | CH25H | TABIMORELIN | inhibitor | ChemblInteractions |  |
| CH25H | CH25H | Definite | CH25H | COBICISTAT | inhibitor | ChemblInteractions |  |
| CXCL2 | CXCL2 | Definite | CXCL2 | ALTEPLASE |  | NCI | 18199827 |
| CXCL2 | CXCL2 | Definite | CXCL2 | BCG VACCINE |  | NCI | 18217952 |
| CXCL2 | CXCL2 | Definite | CXCL2 | DEFEROXAMINE |  | NCI | 17883261 |
| CXCL2 | CXCL2 | Definite | CXCL2 | STAUROSPORINE |  | NCI | 10354507 |
| CXCL2 | CXCL2 | Definite | CXCL2 | BATIMASTAT |  | NCI | 18477053 |
