# Supplementary material for: Identification of biomarkers, immune infiltration landscape, and treatment targets of ischemia–reperfusion acute kidney injury at an early stage by bioinformatics methods
Source: Hereditas. 2022 Jun 4;159:24. doi: 10.1186/s41065-022-00236-x (PMC9167514; doi:10.1186/s41065-022-00236-x)
Supplement: Supplementary file 2 — Additional file 2: Figure S1 Box plots of the gene expression data after normalization. [file 41065_2022_236_MOESM2_ESM.docx]

**Figure S1. Box plots of the gene expression data after normalization.** A: GSE87024, B:GSE34351. The x-axis label represents the gene sample and the y-axis label represents the gene expression values after log2 transformation. The black line in the box plot represents the median value of gene expression.

**Figure S1. Box plots of the gene expression data after normalization.** A: GSE87024, B:GSE34351. The x-axis label represents the gene sample and the y-axis label represents the gene expression values after log2 transformation. The black line in the box plot represents the median value of gene expression.
